# In vitro and in silico studies of the acaricidal and anticholinesterase activities of Randia aculeata seeds against the southern cattle tick Rhipicephalus (Boophilus) microplus

**DOI:** 10.1590/S1984-29612024021

**Published:** 2024-04-29

**Authors:** Aarón Salvador Bustos-Baena, José Luis Bravo-Ramos, Dora Romero-Salas, Sokani Sánchez-Montes, Luis Arturo Ortiz-Carbajal, María Guadalupe Sánchez-Otero

**Affiliations:** 1 Unidad de Investigación y Desarrollo en Alimentos, Instituto Tecnológico de Veracruz, Tecnológico Nacional de México, Veracruz, Veracruz, México; 2 Facultad de Bioanálisis, Universidad Veracruzana, Veracruz, México; 3 Laboratorio de Parasitología, Unidad de Diagnóstico, Rancho Torreón del Molino, Facultad de Medicina Veterinaria y Zootecnia, Universidad Veracruzana, Veracruz, México; 4 Centro de Medicina Tropical, Unidad de Investigación en Medicina Experimental, Facultad de Medicina, Universidad Nacional Autónoma de México, México City, México; 5 Facultad de Ciencias Biológicas y Agropecuarias Región Tuxpan, Universidad Veracruzana, Veracruz, México

**Keywords:** Ticks, molecular docking, bioassays, phytochemical, rutin, Carrapatos, acoplamento molecular, bioensaios, fitoquímico, rutina

## Abstract

*Rhipicephalus* (*Boophilus*) *microplus* is a leading cause of significant economic losses in the livestock industry, and tick populations have developed multiple forms of resistance to acaricides; therefore, the potential of novel natural bioactive compounds that are effective for targeting ticks must be addressed. The aim of this study was to evaluate the acaricidal and anticholinesterase activities of *R. aculeata* seeds and to identify naturally occurring compounds that potentially inhibit anticholinesterase through *in silico* docking. The acaricidal activity of the extract of *R. aculeata* seeds against larval and adult *R. microplus* ticks was assessed through immersion tests. Inhibition of anticholinesterase activity was measured spectrophotometrically. Extracts of *R. aculeata* seeds showed activity against larvae and engorged females of *R. microplus,* and a reduction in the reproductive index were also observed. Rutin, chlorogenic acid, quercetin, and epicatechin exhibited noteworthy interactions with the active site residues of RmAChE. These findings could significantly contribute to the exploration of novel natural products that can potentially inhibit RmAChE and could be used in the development of new acaricides for tick control.

## Resumo

*Rhipicephalus microplus* é uma das principais causas de significativas perdas econômicas na indústria pecuária, e as populações de carrapatos desenvolveram diversas formas de resistência a acaricidas. Portanto, é necessário abordar o potencial de novos compostos bioativos naturais eficazes no combate aos carrapatos. O objetivo deste estudo foi avaliar as atividades acaricida e anticolinesterase das sementes de *R. aculeata* e identificar compostos naturalmente ocorrentes que possam inibir a anticolinesterase por meio de *docking in silico*. A atividade acaricida do extrato de sementes de *R. aculeata* contra larvas e fêmeas adultas de *R. microplus* foi avaliada por meio de testes de imersão. A inibição da atividade anticolinesterase foi medida espectrofotometricamente. Extratos de sementes de *R. aculeata* apresentaram atividade contra larvas e fêmeas ingurgitadas de *R. microplus*, sendo observada também uma redução no índice reprodutivo. Rutina, ácido clorogênico, quercetina e epicatequina mostraram interações notáveis com os resíduos do sítio ativo de RmAChE. Esses resultados podem contribuir significativamente para a exploração de novos produtos naturais que potencialmente inibem RmAChE e poderiam ser utilizados no desenvolvimento de novos acaricidas para o controle de carrapatos.

## Introduction

*Rhipicephalus microplus*, commonly known as the cattle tick, holds significant economic importance as an ectoparasite affecting livestock, primarily in tropical and subtropical areas. This tick species has numerous detrimental impacts on cattle, including blood loss, decreased weight gain, and reduced milk production. Furthermore, it can transmit pathogens such as *Babesia bovis*, *Babesia bigemina* and *Anaplasma marginale* (Rodriguez-Vivas et al., 2018). The primary method for controlling tick infestations is the application of synthetic chemical acaricides; however, this strategy has been linked to other challenges, such as the resistance selection in different tick populations, environmental concerns, and potential residue contamination in meat and milk (Tabor et al., 2017). To address these problems, researchers have been exploring the use of new natural bioactive compounds, which offer improved efficacy and selectivity for targeting ticks (Jain et al., 2020). In the pursuit of discovering new compounds, *in silico* methods such as molecular docking and homology modeling play important roles since these computational techniques enable the identification of molecules that may have affinity for the molecular targets of parasites (Priyadarshini et al., 2019). The discovery of antiparasitic molecules derived from natural sources is costly and time-consuming; therefore, there is growing interest in employing faster and more efficient methods, including the promising approach of integrating *in silico* (computational) and *in vitro* (laboratory) investigations, which has shown feasibility in characterizing potential new acaricides. By utilizing these methodologies, valuable insights can be gained prior to conducting *in vivo* (animal-based) studies, providing valuable guidance in the development of effective treatments (Xiong et al., 2021). *Randia aculeata* L. (Rubiaceae), also known as “crucetillo,” is a small fruit tree with spiny fruits native to the America. The fruit of this tree, widely used by the Mexican population, serves various purposes, including as an antivenom agent and for treating conditions such as diabetes, cancer, inflammation, and pain (Borges et al., 2021). Phytochemical studies of *R. aculeata* have shown the presence of bioactive compounds such as 4-hydroxybenzoic acid, quercetin, chlorogenic acid, caffeic acid, 4-coumaric acid, scopoletin, ferulic acid, rutin, kaempferol, gallic acid, and 2,4-dimetoxi-6-methylbenzoic acid (Juárez-Trujillo et al., 2018). Furthermore, studies have demonstrated the pharmacological properties of several components of *R. aculeata* fruit, such as the pulp, seeds, and shells; these properties include antioxidant, anti-inflammatory, acaricidal and antibacterial activities (Pérez-Espinosa et al., 2003; Gallardo-Casas et al., 2012; Bravo-Ramos et al., 2021; Martínez-Ceja et al., 2022). Another relevant biological property linked to secondary metabolites is their ability to interact with enzymes, including acetylcholinesterase (AChE). AChE is a neural enzyme that is a known molecular targets of ticks. The core role of AChE involves the degradation of the neurotransmitter acetylcholine. The inhibition of AChE in ticks culminates in the extended stimulation of neural pathways, inducing neuromuscular paralysis and ultimately leading to the demise of the tick (Temeyer et al., 2013). Numerous studies have provided evidence through *in silico* and *in vitro* analyses of the efficacy of secondary metabolites against AChE from *R. microplus* (Nasreen et al., 2020; Silva et al., 2021; Cerqueira et al., 2022; Malak et al., 2022). In *R. microplus*, three genes (BmAChE1, BmAChE2, and BmAChE3) encode AChEs, all of which are likely involved in synaptic transmission (Kim & Lee, 2018). Among these enzymes, BmAChE1, the first cholinesterase described in *R. microplus*, exhibits a greater affinity for acetylcholine (Temeyer et al., 2010). Given the demonstrated efficacy of secondary metabolites against insects and other arthropods, it is possible that the synergistic action of these compounds may also make *R. aculeata* effective against arthropods in livestock. Thus, the aim of this study was to evaluate the acaricidal and anticholinesterase activities of *R. aculeata* seeds and to identify which naturally occurring compounds serve as AChE inhibitors through *in silico* docking.

## Materials and Methods

### Botanical material and extract preparation

The fruits of *R. aculeata* were purchased from a local market in Veracruz, Mexico. The taxonomic validation was confirmed by an expert at the Herbarium of the Facultad de Ciencias Biológicas y Agropecuarias, Universidad Veracruzana, who assigned the registration number JLBT2 (VER). Subsequently, the fruits were subjected to washing, and the various components were segregated to isolate the seeds. These seeds were thoroughly cleaned, air-dried for a period of 7 days at room temperature, and subsequently pulverized using a manual grinder. The extraction process was carried out through maceration of the seed material at room temperature with a 1:10 (w/v) proportion of ethanol (Sigma-Aldrich)-water (80:20). Over a span of 3 days, the contents were allowed to settle, and the solvent was collected and subjected to filtration to eliminate solid residues. The extract was subsequently concentrated under vacuum conditions using a Rotavapor R-300 (Buchi®) set at 26 °C. Ultimately, the extract was subjected to lyophilization and stored for further use.

### Tick collection

Engorged adult females of *R. microplus* were manually collected from naturally infested cattle (*Bos indicus* x *Bos taurus*) that were free of acaricide treatments for 30 days before tick collection in a farm located in the municipality of Las Choapas, Veracruz, México where instances of unsuccessful acaricide treatments had been reported by the farm owner. Following collection, the ticks were cleaned with water to remove dirt and then dried using absorbent paper. For the oviposition process, ticks were moved to Petri dishes with holes in the lids to allow proper air circulation. The Petri dishes were then placed under laboratory conditions at a temperature of 28 ± 1 °C and a relative humidity of 85% until oviposition. Then, the egg masses were carefully transferred to glass vials and kept under similar incubation conditions for 20 days to facilitate hatching. After the eggs hatched, the larvae that emerged from the eggs (2 weeks following oviposition) were subjected to larvicidal and anticholinesterase assays. The remaining ticks were subjected to the adult immersion test (AIT).

### Evaluation of acaricide activity: Larval Immersion Test (LIT)

Approximately 100 larvae of *R. microplus* were positioned within 3-mL plastic syringes following the procedures outlined by Silva et al. (2009). The concentrations of hydroethanolic extract derived from *R. aculeata* seeds were determined based on prior research, as detailed in the study of Bravo-Ramos et al. (2021). This study involved the preparation of extracts at multiple concentrations (0.31, 0.62, 1.25, 2.5, 5, and mg mL^-1^) in 70% ethanol. To serve as controls, distilled water and 70% ethanol were employed as negative controls, while amitraz at a 0.0002% concentration was utilized as a positive control. Subsequently, the syringes containing the larvae were immersed in solutions containing various extract concentrations and control substances for 5 minutes. Upon completion of the immersion process, the excess liquid was removed, and the syringes housing the larvae were incubated at a temperature of 26 ± 2 °C and accompanied by a relative humidity above 80% for a span of 24 hours. After the incubation interval, the larvae were stimulated through exposure to a light source, and those that exhibited no movement were considered deceased. To determine larval mortality, Abbott's (1925) formula was applied for correction (% test mortality -% control mortality/ 100 - control mortality x 100). The bioassays were performed in sextuplicate for each extract concentration.

### Adult Immersion Test (AIT)

AITs were performed as described by Sharma et al. (2012). In each replicate (three per concentration), a group of 10 females with similar weights were immersed for 5 minutes in 10 mL of extract solution at each concentration (0.31, 0.62, 1.25, 2.5, 5, and 10 mg mL^-1^). The positive control used was Amitraz®(MSD, USA) at a concentration of 0.0002%, while the negative control consisted of a diluent containing 1% ethanol (Sigma–Aldrich, Germany). and 0.02% Triton X-100® (Sigma–Aldrich, Germany). After the immersion process, engorged females were dried on paper towels and placed dorsally in Petri dishes using two-sided tape. The Petri dishes were then kept in a BOD chamber (Sigma–Aldrich, Germany). at a temperature of 27 ±1 °C and a relative humidity above 80%. The ticks were examined daily using a stereoscope (Olympus SZ51®) and mortality counts were recorded. Dead ticks were identified based on indicators such as hemorrhagic skin lesions, cuticular darkness, and the absence of Malpighian tube movement. After a period of 14 days, the eggs laid by the engorged females were weighed and transferred to labeled tubes, which were subsequently sealed. These tubes were then placed in an incubator under the same environmental conditions for larval hatching and were visually assessed after 16 days of incubation. The results were read by a single technician who was not informed of the treatment details to prevent biased estimations following the procedure outlined by Drummond et al. (1973) and Figueiredo et al. (2018). This approach ensured that the evaluation of the outcomes remained impartial and objective.

The egg production index (EPI), reduction in oviposition (RO), reproduction efficiency index (REI) and efficiency of the extract (EP) were calculated according to the following Formulas 1 to 4 (Bennet, 1974; Roulston et al., 1968; Drummond et al., 1973):


$$EPI\;\left(\%\right)=\;\left(weight\;of\;eggs/weight\;of\;engorged\;female\right)\times 100$$ (1)

$$RO\;\left(\%\right)\;=\;\left[\left(EPI\;control\;group-EPI\;experimental\;group\right)/EPI\;control\;group\right]\;\times 100$$ (2)

$$REI=\left(egg\;mass\;weight\times \;\%\;egg\;hatching/engorged\;female\;weight\right)\times 20,000$$ (3)

$$EP\;\left(\%\right)=\;\left[\left(REI\;control-REI\;treated\right)/REI\;control\right]\;\times 100$$ (4)

### Evaluation of anticholinesterase activity

The procedure began by macerating 100 mg of one-week-old *R. microplus* larvae using a mortar and pestle for 5 minutes. Maceration took place in cold 50 mM phosphate buffer (pH 7.0) with a volume ratio of 1:10 containing 0.5% Triton-X 100 (Sigma–Aldrich, Germany). Following maceration, the homogenate was centrifuged at 2500 × g for 10 minutes at 4 °C. The resulting supernatants were collected and utilized as the enzyme source for subsequent analysis. The activity of acetylcholinesterase (AChE) was assessed with slight modifications to the colorimetric assay described by Ellman et al. (1961). Supernatant aliquots containing *R. microplus* acetylcholinesterase were mixed with *R. aculeata* seed extract at various concentrations (0.31, 0.62, 1.25, 2.5, 5, and 10 mg mL^-1^) and diluted in deionized water. The negative control consisted of deionized water. To initiate the reaction, acetylthiocholine iodide (1 mM) (Sigma–Aldrich, Germany) was utilized as a substrate. The chosen concentrations of the extract were based on previous studies conducted by Bravo-Ramos et al. (2021). Subsequently, the absorbance of the samples was measured at 412 nm (Microplate Reader, Biochrom®).

The percentage of inhibition was calculated as follows:

Inhibition % = (negative control – blank) – (experiment – blank of the experiment) × 100/(negative control – blank). To ensure accuracy and reliability, each sample was subjected to triplicate testing.

### Statistical analysis

The data were analyzed by one-way ANOVA, followed by Tukey’s test to separate means, using STATISTICA v.10 software (α =0.05). The dose–response mortality data were used to estimate the LC_50_ and LC_90_ ﻿by probit analysis and their respective confidence intervals (95% CI) for the extract using ﻿Stata Graphics v.18 software.

### *Rhipicephalus microplus* RmAChE protein sequence

The AChERm model of *R. microplus* was obtained from the UniProt database (https://www.uniprot.org/) with the accession code A0A0F6P2D6. The active site consists of Ser256, His494 and Glu 381, corresponding to carboxyl ester hydrolases.

### *In silico* structural analysis of AChERm

The 3D model of AChERm, consisting of 595 amino acids, was obtained using AlphaFold artificial intelligence with 96% confidence (pLDDT). The active site is internalized in the cavity and consists of 51 amino acids. The ProteinPlus DoGSiteScore server (https://proteins.plus/) calculates and locates the different cavities with ligand binding potential of a structure by the difference of Gaussians (DoG) method. The volume of the cavity containing the active site was 1040 Å^3^, the surface area was 960 Å^2^, and the DoGSiteScore was 0. 85, consisting of the amino acids GLN122, VAL123, LEU124, ASP125, SER132, GLY133, SER134, MET136, TRP137, ASN138, ALA139, TRP171, ILE172, TYR173, GLY174, GLY175, GLY176, TYR178, SER179, GLY180, THR181, LEU184, VAL186, TYR187, GLU255, SER256, ALA257, SER282, TRP289, VAL341, PHE343, GLU381, TRP384, PHE385, TYR388, PHE454, SER481, GLN483, ASN484, PRO485, TRP486, ILE493, HIS494, GLY495, GLU496, VAL498, PRO499, PHE500, GLU504, TYR510 and TYR512.

### Ligands and protein preparations

We identified a total of 12 compounds in the extract of *R. aculeata* seeds through HPLC/MS analysis that can potentially act as acetylcholinesterase inhibitors (chlorogenic acid, vanillinic acid, p-coumaric acid, caffeic acid, rutin, quercetin, epicatechin, 4-hydroxybenzoic acid, vanillin, 2,4-dimethoxy-6-methylbenzoic acid, scopoletin and ferulic acid) (Bravo-Ramos et al., 2023).

The structures of the compounds were obtained from the PubChem database (https://pubchem.ncbi.nlm.nih.gov/) by performing an initial 300-step structure optimization with the JSmol server, correcting charges, and steric hindrances of the molecule. Avogadro (1.2.0) was applied to add hydrogens with respect to the pH to be used for the simulation, which was 7.4. A second minimization of the energy of the molecules was performed using the MMFF94 force field, and the Auto Optimize tool continuously optimized the molecular geometry through molecular mechanics. The optimization stops once the energy change (dE) is equal to zero or approximately zero, where E is the lowest energy value.

### Molecular docking

For molecular docking, the AChE1 model was parameterized in UCSF Chimera software using the DocPrep function with the AMBER ff14SB/AM1-BCC force field and using the minimization function. Substrates were parameterized with the AMBER ff14SB/Gasteiger force field. Molecular docking was performed with UCSF Chimera/AutoDockVina, and the box was designed by spanning the cavity where the active site is located (6, 1.2, -0.4) and by length (20, 16.4, 20.2).

## Results

### *In vitro* larvicidal activity

**Figure 1:**
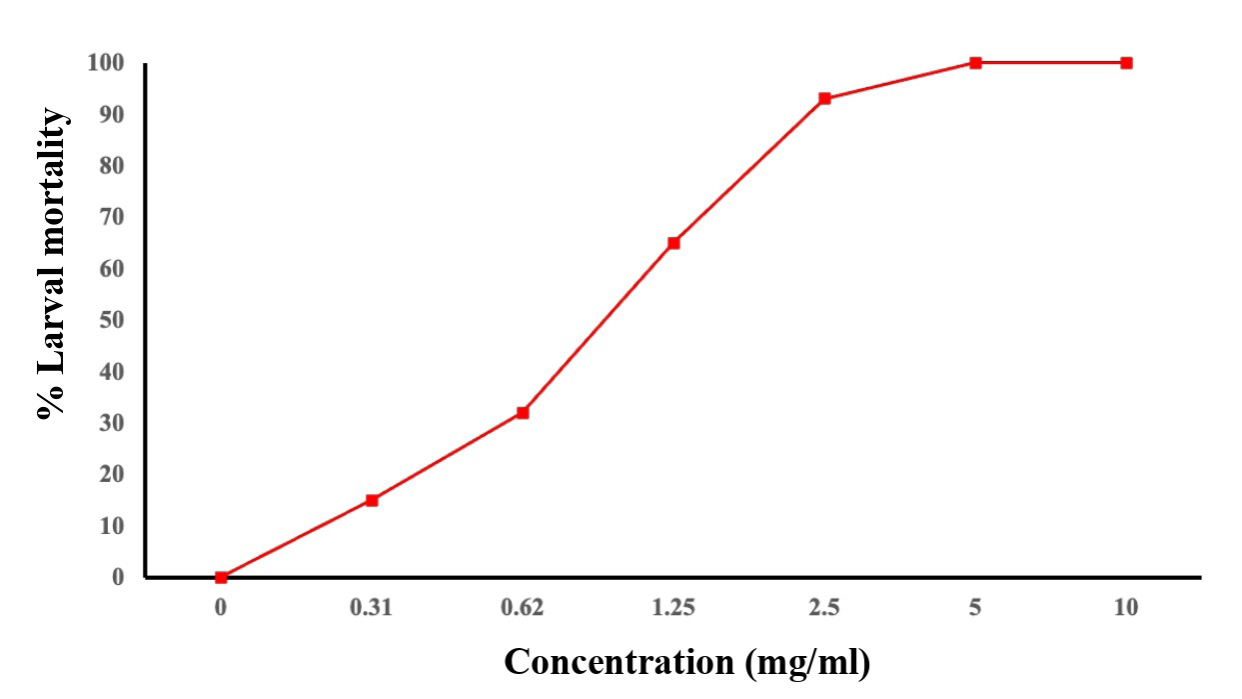
Dose-dependent mortality against *R. microplus* larvae of extract of *R. aculeata* seed.

*R. aculeata* seed extract exhibited noteworthy larvicidal activity, causing more than 50% mortality in the larvae at the concentrations tested, except for 0.32 and 0.61 mg mL^-1^ after 24 hours (Figure 1). This level of mortality was comparable to that induced by the positive control Amitraz. The concentrations of the extracts were assessed to determine the lethal concentrations, which were subsequently subjected to probit analysis. ﻿The LC_50_ and LC_90_ values of the extracts are shown in Table 1.

**Table 1:** LC_50_ and LC_90_ (﻿mg mL^-1^; in parentheses the 95% confidence limits) for mortality of *R. microplus* larvae and engorged females exposed to *R. aculeata* seed extract.

Tick stage	LC_50_	LC_90_
Larvae	1.10 (0.87- 2.34)	9.6 (8.2- 10.4)
Engorged female	4.8 (3.2-7.8)	17. 6 (15.8-25.4)

### Adult Immersion Test (AIT)

﻿Table 2 displays the percentages of mortality and changes in reproductive parameters observed for the *in vitro* effectiveness of *R. aculeata* seed extract against engorged females of *R. microplus* ﻿when tested at 0.31–10 mg mL^-1^. Egg hatching was effectively inhibited by higher concentrations (2-5-10 mg mL^-1^) of extract. However, the other concentrations (0.31-1.25 mg mL^-1^) only partially hindered egg hatching, although the newly hatched larvae were unable to survive and died shortly after hatching. Furthermore, a higher concentration (10 mg mL^-1^) of extract had a noteworthy impact on adult engorged ticks, leading to high mortality. Additionally, there was a considerable reduction in the mass of eggs laid by the ticks treated with all extract concentrations compared to those in the control group. Consequently, this significant reduction in the egg laying capacity of the ticks resulted in a marked decrease in the reproduction efficiency index. ﻿The LC_50_ and LC_90_ values of the extracts are shown in Table 1.

**Table 2:** Effect of extract of *Randia aculeata* seed on mortality (%), egg mass (g), egg production index (EPI, %), reduction in oviposition (RO, %), reproduction efficiency index (REI), larval hatching (%) and efficacy of the extracted product (EP, %) in engorged females of *R. microplus*.

		Mortality %	egg mass (g)	EPI (%)	RO (%)	REI	Larval hatching (%)	EP (5)
Negative control	Control	0.0	0.78 ± 0.04	42.9 ± 1.2	0.0	28.5 ± 2.1	100	0.0
	0.31	0.0^a^	0.98 ± 0.02	39 ± 1.9	8.1 ± 0.8	26.4 ± 1.0	89	7.3 ± .4
	0.62	10.6 ± 3.0^b^	0.79 ± 0.02*	35.6 ± 0.8	17. ± 1.2	22.3 ± 1.3*	74	21.7 ± 1.2
*R. aculeata* (seed)	1.25	34.6 ± 5.4^c^	0.57 ± 0.03*	32.6. ± 0.1	24.6 ± 1.1	17.5 ± 1.3*	42	38.5 ± 1.3
	2.5	47.5 ± 6.7^d^	0.48 ± 0.03*	24.5 ± 0.5	42.8 ± 1.2	12.7 ± 1.5*	28	55.4 ± 1.8
	5.0	53.0 ± 2.9^d^	0.35 ± 0.02*	19.5 ± 0.4	54.5 ± 0.5	5.3 ± 0.3*	19	81.4 ± 2.3
	10.0	72.0 ± 5.4^e^	0.32 ± 0.02*	15.1 ± 0.5	64.1 ± 0.4	4.3 ± 0.3*	12	84.9 ± 1.5

* Indicates significant differences from the negative control *P<0.05*.

### *In vitro* anticholinesterase activity

**Figure 2:**
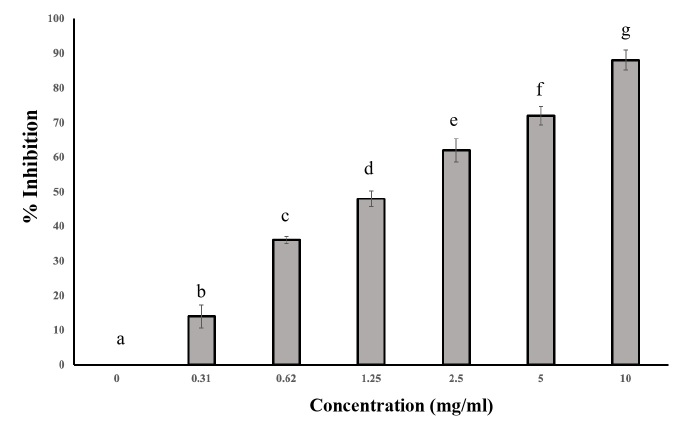
*In vitro* inhibition (mean ± standard deviation) of the acetylcholinesterase enzyme from *R. microplus* larvae exposed to *R. aculeata* seed. Different letters in the columns indicate a statistically significant difference (*P*< 0.05).

The effect of the inhibition of AChE activity by *R. aculeata* seed extract is shown in Figure 2. The results demonstrated that this extract reduced AChE activity. Compared with those in the control group, the inhibition of *R. microplus* was significant at all concentrations tested *(P*<0.05).

### Docking studies

**Figure 3:**
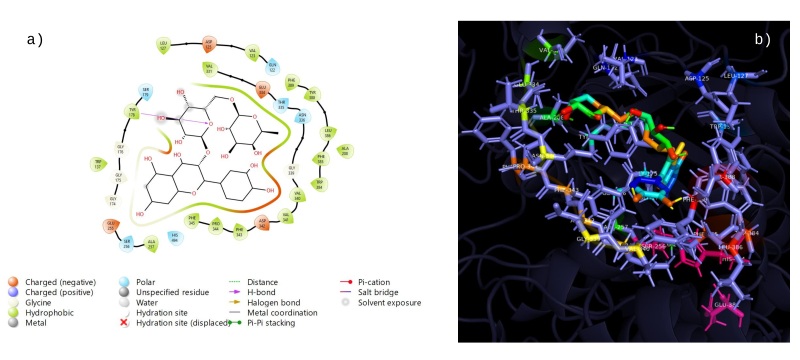
Interactions 2D (a) and 3D (b) of rutin (9) with the active site of *R. microplus* AChE.

**Figure 4:**
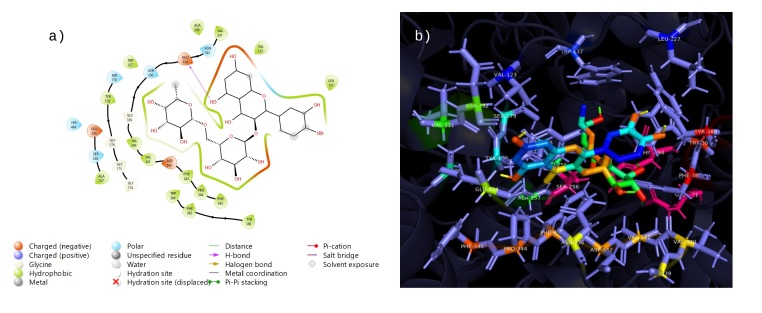
Interactions 2D (a) and 3D (b) of chlorogenic acid (4) with the active site of *R. microplus* AChE.

**Figure 5:**
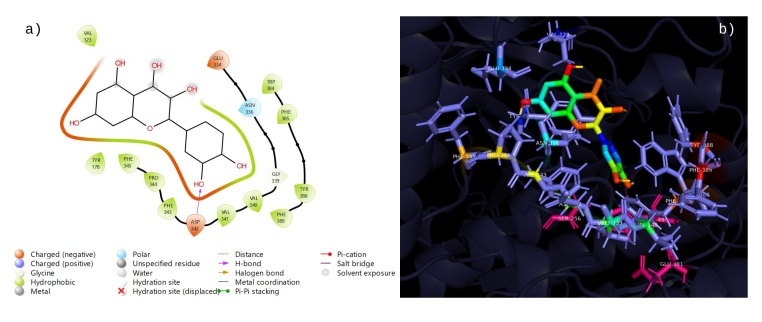
Interactions 2D (a) and 3D (b) of quercetin (8) with the active site of *R. microplus* AChE.

**Figure 6:**
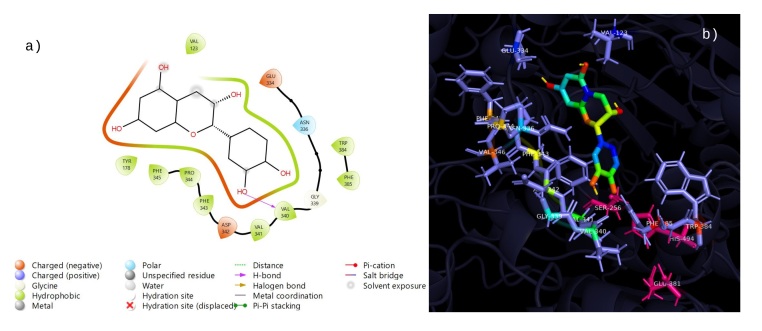
Interactions 2D (a) and 3D (b) of epicatechin (5) with the active site of *R. microplus* AChE.

The twelve compounds identified in the phytochemical study of the ethanolic extract from *R. aculeata* seeds were chlorogenic acid, vanillinic acid, p-coumaric acid, caffeic acid, rutin, quercetin, epicatechin, 4-hydroxybenzoic acid, vanillin, 2,4-dimethoxy-6-methylbenzoic acid, scopoletin and ferulic acid. Additionally, ﻿we summarize the phytochemical docking scores for the AChERm active site (Supplementary File 1: Table S1). According to these results, rutin (9) is the most potent inhibitor, with a docking score of -8.9 kcal/mol. The binding energies of chlorogenic acid (4) (-8.6 kcal/mol), quercetin (8) (-7.9 kcal/mol) and epicatechin (5) (-7.5 kcal/mol) were among the highest among those of all the analyzed compounds; therefore, these compounds exhibit better docking ability with the crystal structure of AChERm. Molecular docking of Rutin (9) is depicted in Figure 3. At least three hydrogen bonds are established with TYR178, ASN336 and HIS494; interestingly, TYR178 also establishes a couple of π-π stacked and π-alkyl interactions favoring the complexation of Rutin with the enzyme. Additionally, one of the aromatic rings of Rutin establishes a π-alkyl interaction with VAL123. The interaction of chlorogenic acid (4) with the active site of AChERm is depicted in Figure 4. This compound establishes an interaction π-anion with GLU225 and π-alkyl interactions with PHE385, TRP384, and VAL498, which allows the establishment of a stable complex with the enzyme. Additionally, chlorogenic acid is bound to GLY175, GLY176 and TYR178 through conventional hydrogen bonding, with an unfavorable bond with SER 256 and PHE 343. The results of the molecular docking of quercetin (8) with AChERm are depicted in Figure 5. There are three conventional hydrogen bonds between quercetin and GLY339, VAL340 and ASP342, and a π-donor hydrogen bond is observed with ASN336. Several π interactions are observed: π-alkyl (VAL123; PRO344) and π-π stacked and π-π shaped interactions with TYR178 and PHE343. The interaction between epicatechin (5) and AChERm is shown in Figure 6. TYR178 and PHE 343 establish π-π stacked and π-π shaped interactions with epicatechin. There is a π-alkyl interaction with TYR178, and another two π alkyl groups are established with VAL123 and PRO344, all of which contribute to the formation of a complex with high stability. Additionally, there are two hydrogen bonds with VAL340 and GLU334.

## Discussion

*In vitro* screening plays a pivotal role in compound identification and selection for advancing the development of novel products targeting *R. microplus* control. The current study underscores the substantial acaricidal efficacy of *R. aculeata* seed extract against *R. microplus* ticks.

The larval phase serves as a common choice for assessing the acaricidal potential of plant-derived compounds *in vitro*. However, compound effectiveness can fluctuate based on the tick's developmental stage (Rosado-Aguilar et al., 2017). In adult ticks, the presence of a more prominent waxy layer could diminish efficacy by confining compounds within the wax matrix (Adenubi et al., 2018). Discrepancies may also stem from factors such as the plant's specific production profile or the solvent employed, as well as the developmental stage of the plant at the time of collection. Notably, the current study established a pattern of enhanced tick mortality at lower concentrations after *in vitro* treatment. This outcome resonates with the findings of Bravo-Ramos et al. (2021), who highlighted the dose-dependent and exposure time-dependent mortality effect of *R. aculeata* seed extract. Furthermore, within our study, we noted heightened anticholinesterase activity in the *R. aculeata* seed extract. This observation implies a potential connection between larvicidal activity and this specific mechanism of action. Consequently, *in silico* experiments are essential for corroborating the indications of acaricidal activity highlighted in this study for the investigated plant.

Flavonoids have been extensively studied as bioactive compounds with various biological properties, including antioxidant and antidiabetic effects. With respect to human AChE inhibitory activity, there is increasing interest in the application of AChE in preventing and treating Alzheimer disease (Khan et al., 2009; Mani et al., 2023) and, more recently, in the application of AChE from arthropods due to the resistance phenomena among diverse tick infestations, such as *R. microplus*. According to the *in silico* studies, among the compounds identified in the phytochemical study of *R. aculeata* seeds, four had higher docking scores: rutin, chlorogenic acid, quercetin and epicatechin.

Rutin is a flavonoid glycoside synthesized in plants with high antioxidant capacity. Rutin has antibacterial, anti-inflammatory, antiviral and many other properties. Docking studies of rutin interactions with AChE from different species of *R. microplus* have been reported. Yan et al. (2018) compared the AChE inhibitory effects of forsythiaside and rutin and revealed that rutin was located in the active site gorge, where it formed hydrogen bonds and hydrophobic interactions. The thermodynamic parameters indicated that hydrophobic forces played a major role. In relation to AChE from *R. microplus*, rutin has been proven to establish stable interactions with a binding energy of -6.8 kcal/mol, consisting of hydrogen bonds and different π interactions, such as π-T-shaped and π-alkyl interactions, which favor stable complexes (Chen et al., 2022). In this work, we found a –8.9 kcal/mol docking score, the highest of which was calculated for the 12 compounds found in the *R. aculeata* extract, with several interactions, including π-π stacking and π-alkyl interactions and hydrogen bonds, which is consistent with the high docking score. A similar docking score for the interaction between rutin and AChEERm was reported by Malak et al. (2022), who also reported an interaction with the TYR178 amino acid.

Chlorogenic acid is present in a wide array of medicinal plants (Li et al., 2010). Chlorogenic acid is the isomer 5-O-caffeoylquinic acid and has been extensively studied due to its early availability in a pure crystal form compared with other isomers. This compound can be consumed in diverse foods, such as coffee beans, artichoke, and sunflower seed kernels. Chlorogenic acid has been proven to promote a broad range of health benefits and biological effects on conditions associated with oxidative stress, such as metabolic syndrome and hepatic steatosis, and has several neuroprotective effects (Lu et al., 2020). The insecticidal of chlorogenic acid activity has been demonstrated against whitefly (*Bemisia tabaci*), with no observed cross-resistance to other pesticides (Wang et al., 2023). Docking studies of human AChE have demonstrated that chlorogenic acid might have high inhibitory activity due to the large number of functional groups interacting with key amino acids on human AChE (Isık & Beydemir, 2021). In this study, the major component of the *R. aculeata* seed extract, chlorogenic acid, exhibited an affinity for AChERm (-8.6 kcal/mol), which might be related to the π interactions that favor the establishment of a stable complex with the enzyme. In contrast, Chen et al. (2022) calculated the affinity energy between AChE from *Electrophorus electricus* and chlorogenic acid and compared it with that of other active compounds from *Polygonatum sibricum* (isoquercetin and scopoletin), resulting in a lower affinity energy than that of the other compounds.

Quercetin (3,3’,4’,5,7-pentahydroxyflavone) is a flavonoid and the aglycone form of rutin. Quercetin is commonly found in edible vegetables such as onions, apples, and berries, and it is also present in medicinal plants such as *Ginkgo biloba*. Several biological acitivites of quercetin, such as antibacterial, antifungal and antiviral activities, have been described, in addition to its use as an antidepressant, in the treatment of cancer, in the treatment of allergies and as a neuroprotective agent (Rajesh & Dhanaraj, 2023). Liao et al. (2022) investigated the inhibitory mechanism of quercetin on human acetylcholinesterase to demonstrate its neuroprotective effects on oxidative stress injury in PC12 cells. The authors reported π-π stacking, hydrogen bonding and van der Waals forces between quercetin molecules. Molecular docking with quercetin and AchERm has been explored by Cerqueira et al. (2022), who evaluated the Gridscore (kcal/mol) of quercetin and other flavonoids and compared it to that of eserine, a synthetic acaricide, both of which resulted in higher values than those of the other compounds. Similarly to our results, the authors reported parallel and perpendicular hydrogen bond and p-stacking interactions; we observed several π interactions, such as π-alkyl interactions (VAL123; PRO344), π-π stacking and π-π shapes, with TYR178 and PHE343.

Epicatechin is a flavanol present in vegetables, fruits, cereals, cocoa, and tea. Epicatechin has protective effects on human health through its high antioxidant and anti-inflammatory activities that have been associated with nervous system protection. To our knowledge, there are no docking analyses of epicatechin or AchERm, but in the docking analysis of several phytochemicals and human AChE conducted by Mani et al. (2023), epicatechin was shown to have a binding affinity of –9.6 kcal/mol higher than that of most of the studied ligands. The authors reported that epicatechin established hydrogen bonds and hydrophobic interactions with residues in the active site.

## Conclusion

*R. aculeata* exhibited larvicidal activity against *R. microplus in vitro*, achieved by inhibiting the acetylcholinesterase enzyme. Moreover, the docking scores observed in this study indicate favorable interactions between the active compounds from *R. aculeata* seeds and the selected tick target. The ligands rutin, chlorogenic acid, quercetin, and epicatechin possess highly desirable drug-like properties and pharmacokinetic characteristics. Consequently, this research highlights the potential of *R. aculeata* to effectively combat the cattle tick *R. microplus*. This discovery may lead to the development of cost-effective, safe, and environmentally friendly herbal acaricides. Future studies will be carried out to assess the acaricidal activity of these compounds under *in vivo* conditions.

## Supplementary Material

Supplementary material accompanies this paper.

Table S1 Docking scores for various *R. aculeata* seed compounds.

This material is available as part of the online article from https://doi.org/10.1590/S1984-29612024021
