# Supplementary material for: In vitro and in silico studies of the acaricidal and anticholinesterase activities of Randia aculeata seeds against the southern cattle tick Rhipicephalus (Boophilus) microplus
Source: Rev Bras Parasitol Vet. 2024 Apr 29;33(2):e001524. doi: 10.1590/S1984-29612024021 (PMC11065400; doi:10.1590/S1984-29612024021)
Supplement: Table S1 [file rbpv-33-2-e001524-Suppl.pdf]

Table S1. Docking scores for various *R. aculeata* seed compounds.

Number	Compound name	Compound structures	Docking score kcal/mol	RMSD	Amino acids residue interactions
1	2,4-Dimethoxy-6-methylbenzoic acid	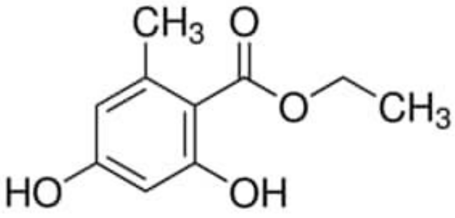	-3.5	0	Trp137, Tyr173, Gly174, Gly175, Gly176, Tyr178, Ser179, Tyr187, Glu255, Ser256, Ala257, Phe343, Trp384, Phe385, His494, Gly495, Glu496 y Val498
2	4-hydroxybenzoic acid	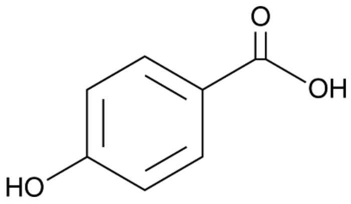	-4.7	0	Trp137, Gly174, Gly176, Tyr178, Glu255, Ser256, Phe343,

					Trp384, Phe385, His494 y Gly495
3	caffeic acid	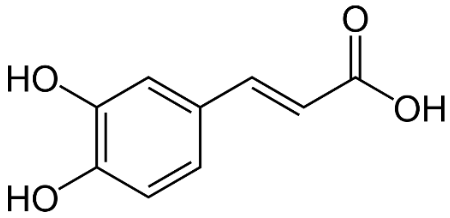	-4.8	0	Trp137, Gly174, Gly175, Gly176, Tyr178, Tyr187, Glu255, Ser256, Phe343, Trp384, Phe385, His494, Gly495 y Val498
4	chlorogenic acid	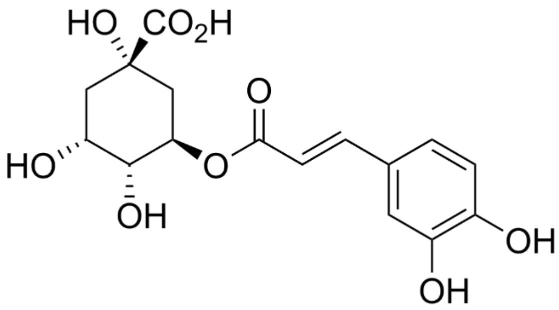	-8.6	0	Trp137, Trp171, Tyr173, Gly174, Gly176, Tyr178, Val186, Tyr187, Glu255, Ser256, Ala257, Trp289,

					Val341, Phe343, Trp384, Phe385, Phe454, His494, Gly495 y Val498
5	epicatechin	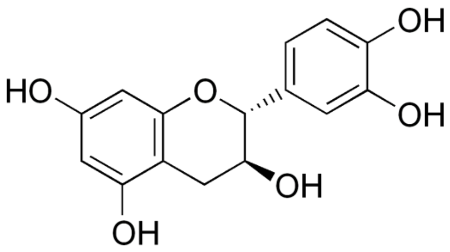	-7.5	0	Val123, Tyr178, Glu178, Glu334, Asn336, Gly339, Val340, Val341, Asp342, Phe343, Pro344, Phe345, Trp384 y Phe385
6	ferulic acid	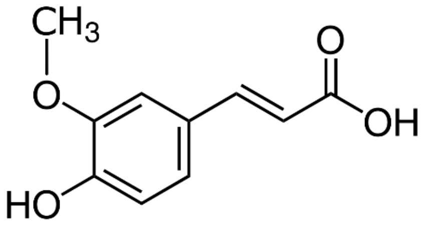	-4	0	Trp137, Gly174, Gly175, Gly176, Tyr178, Ser179, Tyr187, Glu255,

					Phe343, Trp384, Phe385, His494, Gly495 y Val498
7	p-coumaric acid	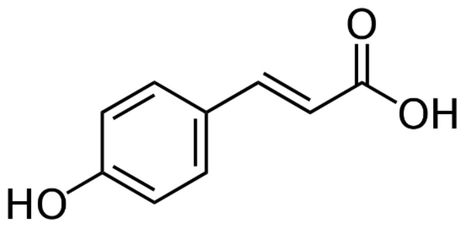	-4.2	0	Trp137, Gly174, Gly175, Gly176, Tyr178, Tyr187, Glu255, Ser256, Phe343, Trp384, Phe385, His494, Gly495 y Val498
8	quercetin	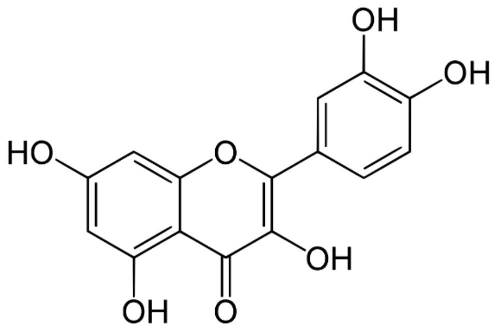	-7.9	0	Val123, Tyr178, Glu334, Asn336, Gly339, Val340, Val341, Asp342, Phe343, Pro344, Phe345,

					Trp384, Phe385, Tyr388 y Phe389
9	rutin	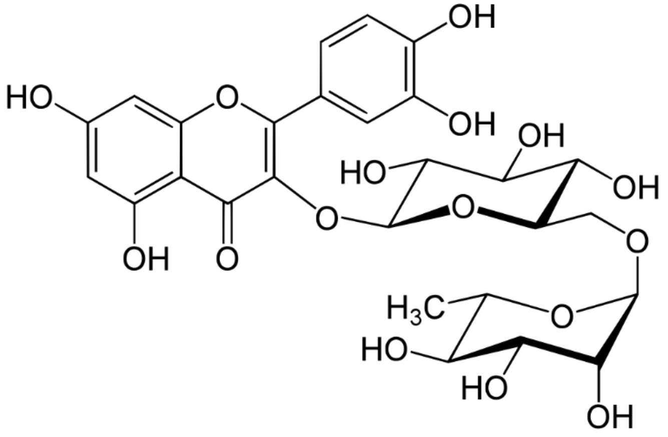	-8.9	0	Val123, Leu127, Trp137, Gly174, Gly175, Gly176, Tyr178, Ser179, Glu255, Ser256, Val331, Asn332, Asn333, Glu334, Asn336, Gly339, Val340, Val341, Asp342, Phe343, Pro344, Trp384, Phe385,

					Tyr388, Phe389 y His494
10	vanillin	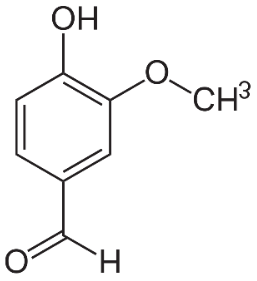	-4.9	0	Trp137, Gly174, Gly175, Tyr187, Glu255, Ser256, Trp384, Phe385, His494 y Gly495
11	vanillinic acid	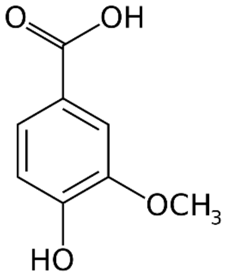	-4.6	0	Trp137, Trp171, Tyr173, Gly174, Gly175, Gly176, Tyr187, Glu255, Ser256,

					Phe343, Trp384, Phe385, His494, Gly495 y Val498
12	scopoletin	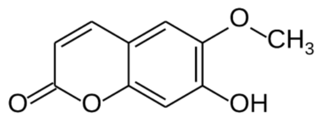	-4.2	0	Trp137, Trp171, Gly174, Gly175, Gly176, Tyr187, Glu255, Ser256, Trp384, Phe385, His494, Gly495 y Val498
